# A global perspective on the issue of access to insulin

**DOI:** 10.1007/s00125-020-05375-2

**Published:** 2021-01-23

**Authors:** David Beran, Maria Lazo-Porras, Camille M. Mba, Jean Claude Mbanya

**Affiliations:** 1 Division of Tropical and Humanitarian Medicine, University of Geneva and Geneva University Hospitals, Geneva, Switzerland; 2 CRONICAS Centre of Excellence in Chronic Diseases, Universidad Peruana Cayetano Heredia, Lima, Peru; 3 MRC Epidemiology Unit, University of Cambridge, Cambridge, UK; 4 Department of Public Health, Faculty of Medicine and Biomedical Sciences, University of Yaoundé 1, Yaoundé, Cameroon; 5 Department of Internal Medicine and Specialties, Faculty of Medicine and Biomedical Sciences, University of Yaoundé 1, Yaoundé, Cameroon

**Keywords:** Developing countries, Diabetes mellitus, Health services accessibility, Insulin, Review

## Abstract

The discovery of insulin in 1921 changed the prognosis for people with type 1 diabetes. A century later, availability and affordability of insulin remain a challenge in many parts of the globe. Using the WHO’s framework on understanding the life cycle of medicines, this review details the global and national challenges that affect patients’ abilities to access and afford insulin. Current research and development in diabetes has seen some innovations, but none of these have truly been game-changing. Currently, three multinational companies control over 95% of global insulin supply. The inclusion of insulin on the WHO’s Prequalification Programme is an opportunity to facilitate entry of new companies into the market. Many governments lack policies on the selection, procurement, supply, pricing and reimbursement of insulin. Moreover, mark-ups in the supply chain also affect the final price to the consumer. Whilst expenses related to diabetes are mostly covered by insurance in high-income countries, many patients from low- and middle-income countries have to pay out of their own pockets. The organisation of diabetes management within the healthcare system also affects patient access to insulin. The challenges affecting access to insulin are complex and require a wide range of solutions. Given that 2021 marks the centenary of the discovery of insulin, there is need for global advocacy to ensure that the benefits of insulin and innovations in diabetes care reach all individuals living with diabetes.

**Supplementary Information:**

The online version contains a slideset of the figures for download, which is available at 10.1007/s00125-020-05375-2.

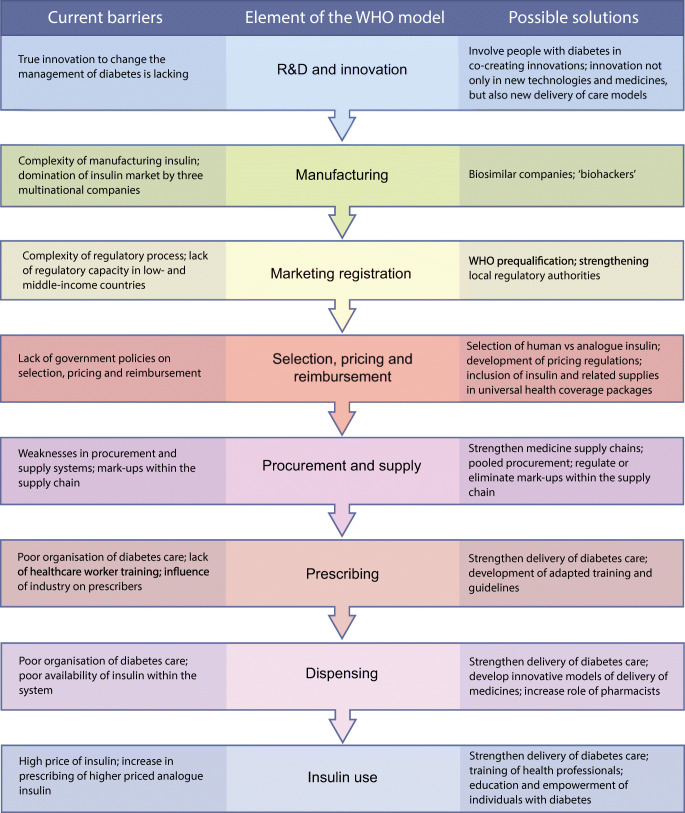

## Introduction

The year 2021 marks a momentous milestone for the diabetes community, with the centenary of the discovery of insulin. Insulin’s discovery by Banting and Best at the University of Toronto (ON, Canada) in 1921 meant that type 1 diabetes went from being a death sentence to a manageable chronic condition. Leonard Thompson was the first person to receive insulin as a treatment for type 1 diabetes in 1922 in Canada. That same year, Elliot Joslin stated, ‘A new race of diabetics [people with diabetes] has come upon the scene’ [1] due to the discovery of insulin. Later, in 1925, in his book ‘A diabetic life’ [2], Robert D. Lawrence declared, ‘Now modern discoveries, particularly insulin, have completely changed the outlook. There is no reason why a diabetic [person] should not, if he can be taught to do so, lead a long normal life’.

However, these initial positive views on the prospects of insulin therapy in the 1920s are in harsh contrast to the reality that many people with diabetes face some 100 years later, specifically owing to lack of access to insulin. Basu et al. [3] found that, globally, in individuals with type 2 diabetes, one in two people had access to the insulin they needed; whilst in sub-Saharan Africa, this number was found to be to only one in seven people, highlighting the impact of poor health systems, and lack of access to insulin and other tools necessary for diabetes management, on the effective delivery of diabetes care.

## Global barriers to insulin access

**Fig. 1:**
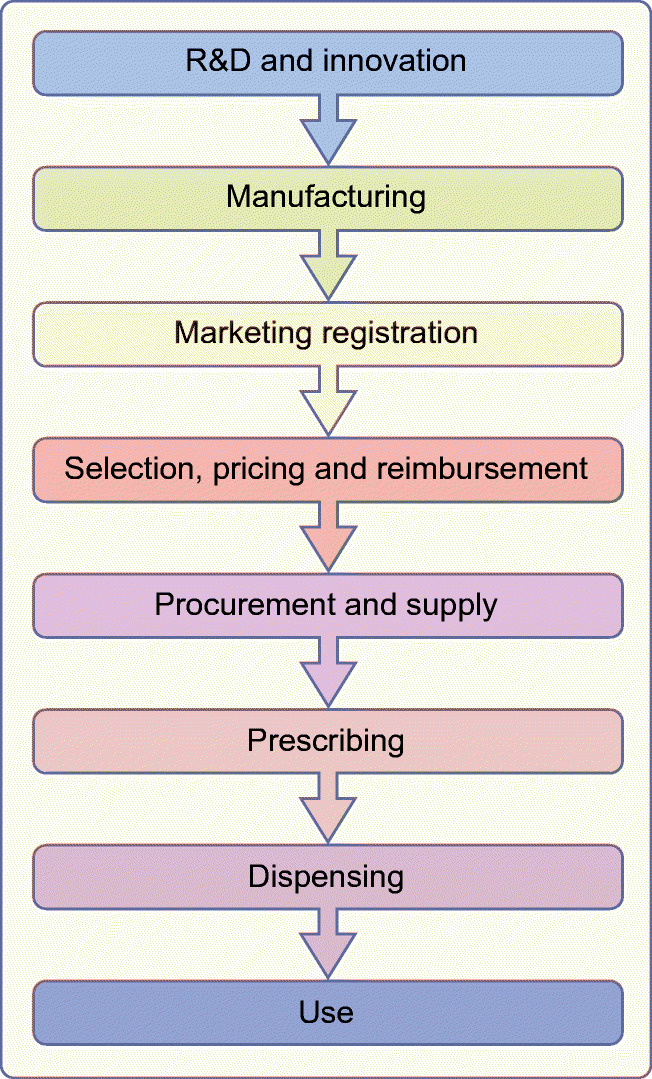
Schematic of WHO framework on understanding the life cycle of medicines [9]. This framework provides the key elements from a medicine’s discovery until it is used by an individual, highlighting key barriers along this pathway. This figure is available as part of a downloadable slideset

In 2016, the former Director-General of the WHO declared that ‘people with diabetes who depend on life-saving insulin pay the ultimate price when access to affordable insulin is lacking’ [4]. A study in 13 low- and middle-income countries (LMICs), published in 2019, found that mean availability of insulin was 55–80% in facilities that should have had insulin available on the day of study [5]. Furthermore, in looking at standardised prices per 10 ml vial of 100 U insulin in this study, the median price governments pay (the government procurement price) for human insulin was shown to be US$5, with analogue (long-acting) insulin being 6.6 times more expensive. Across public and private pharmacies and private hospitals, the median price that an individual has to pay for human insulin is US$9 [5]. Clearly, in LMICs, these prices are out of reach for most individuals; however, the affordability of insulin is also an issue in high-income countries [6–8]. The WHO’s framework on understanding the life cycle of medicines [9] provides a useful model for understanding the complexity of the barriers to access to insulin that are present globally (Fig. 1).

### Research and development and innovation

The first component of the WHO framework [9] is research and development (R&D) and innovation. In a study in 2016, Kaplan and Beall looked at insulin-related patents to assess R&D and innovation and found that no patents existed for human insulin and many patents for analogue insulins expired in 2015 for products already on the market [10]. Most of the patents were filed by large multinational companies, with a few of the patents identified being from companies in China and India. In the USA, more than half of patents were found to be for insulin-delivery devices rather than insulin itself [11].

The transition from animal to human and then analogue insulin is seen as an innovation [12], but one that comes at a higher price [13, 14]. Some innovations in modes of administration of insulin (inhalable or oral insulin) have not shown any success [15, 16]. The promising so-called ‘smart insulin’ or glucose-responsive insulin that would reduce the risk of hypoglycaemia associated with insulin’s use is one innovation that is currently in the pipeline [17].

### Manufacturing

**Fig. 2:**
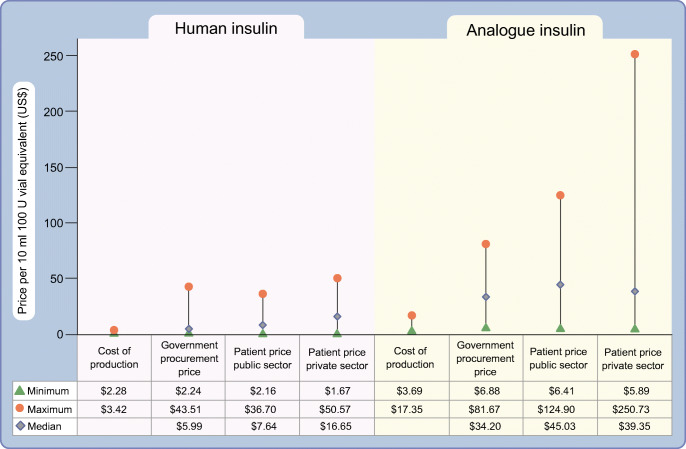
Costs of production and government procurement, and public and private sector patient prices for human and analogue insulin, based on 43 countries. Data for cost of production from [20]; data for government procurement costs and public and private sector patient prices from [60]. This figure is available as part of a downloadable slideset

In looking at the manufacturing of insulin, two factors need to be considered: (1) insulin is a complex biological product that requires specific expertise to manufacture in order to ensure a high quality, safe and efficacious product [6]; (2) at present, the manufacturing of insulin is concentrated in three large multinational companies—Eli Lilly, Novo Nordisk and Sanofi (known as ‘the big three’). Eli Lilly, Novo Nordisk and Sanofi have a 96% share of the insulin market by volume and hold 99% of the market by value [6]. Other insulin manufacturing companies have been identified, including producers in India and China [18], but their current impact on the global market is negligible. In parallel to industrial production, so-called ‘biohackers’ are attempting to provide an open-source method for the production of insulin to possibly lower the costs of production, as well as increase competition [19]. Gotham et al. [20] estimated that the cost of production for human insulin for a 10 ml 100 U vial would be between US$2.28 and US$3.42, whilst the cost of manufacturing most formulations of analogue insulin was slightly higher (US$3.69–6.34), with the exception of insulin detemir, with costs of production of US$13.47–17.35. In comparing the median government procurement prices in different countries with the cost of production, procurement prices were found to range from 1.8 to 2.6 times higher than production costs for human insulin, and 2.0 to 9.3 times higher than production costs for analogue insulin (Fig. 2).

### Marketing and registration

All medicines are required to have marketing authorisation and must be registered by a national regulatory agency before placement on the market. This requires scientific evaluation of the product to ensure that it meets specific standards of safety, efficacy and quality [21]. For biological products, such as insulin, this process is more complex than for chemical entities. For biosimilars, generics of biological products, the process of regulatory assessment is demanding and includes the need for clinical trials [22]. These requirements affect the cost of insulin and the entry of biosimilar products into the market.

In parallel to the complex process that is needed for insulin manufacturers to produce and get their products approved by regulatory agencies, there is also the challenge that many regulatory agencies in LMICs do not have the technical capacity to assess the data in the dossiers prepared for biological products. To address this, the WHO developed the Prequalification Programme in 2001, mainly in response to HIV/acquired immune deficiency syndrome (AIDS), to assess the quality of anti-retroviral products. The WHO announced the launch of prequalification for insulin in November 2019 [23]. This allows any producer of insulin to submit their product for regulatory review by the WHO. Through this process, competition in the insulin market will hopefully be increased whilst ensuring safety and efficacy of products for governments and their populations.

### Selection, pricing and reimbursement

Just because a medicine meets regulatory standards, this does not necessarily mean that it is selected and used. The WHO’s Model Essential Medicines List (EML) is aimed at guiding individual countries with regard to their decisions on their choice of medicines, and only includes guidance on soluble and intermediate-acting insulin in vial form [24]. Although long-acting insulin analogues were put forward for inclusion on this list in 2019, they were not included because the ‘available evidence shows efficacy and safety advantages of analogues compared to human insulin which are insufficiently large to justify the cost differential that continues to exist.’ [25] For analogues to be considered an essential medicine and included in the WHO EML, more evidence is needed on their effectiveness, as well as a decrease in their price [26].

For pricing of medicines, different countries have different policy measures for both the system and individuals [27]. For example, in Europe, these processes include health technology assessments to help determine the ‘therapeutic value’ of a medicine, referencing pricing, value-based pricing (price established based on ‘added therapeutic value’), policies encouraging the uptake of generic products and tendering at different levels of the health system [28]. Such measures do not exist in many LMICs [27] meaning that, as in the USA, prices are free to be set by the ‘market’. This can result in increasing prices of insulin, as has been seen in the USA [29], due to a lack of government control on medicine pricing. Figure 2 shows the prices for government procurement of insulin (per 10 ml vial of 100 U insulin), which range from US$2.2 to US$43.5 for human insulin and US$6.9 to US$81.7 for analogue insulin.

In many LMICs, the price of insulin is paid for in full by an individual [27] or, in some contexts, subsidies are in place [30]. In contrast, in most high-income countries, a variety of government-funded or insurance schemes provide some form of financial protection, either ensuring that insulin is provided for free to the individual or, at least, that the person does not bear the full cost [27, 28].

### Procurement and supply

Pricing policies and approaches taken by governments will affect how medicines are procured. In most LMICs, insulin is purchased via centralised tenders for the public sector [31]. In the private sector, this is done via wholesalers and private pharmacies. Another source of insulin includes donation programmes, such as Life for a Child or Novo Nordisk’s Changing Diabetes in Children [32]. Although these initiatives have had a positive impact for individuals with type 1 diabetes, they have not been integrated into the formal health system, which raises issues of sustainability [32].

Once insulin is procured, dependent on the sector and health system, the price difference between the government procurement price and patient price might increase due to import tariffs, storage costs, transportation costs, dispensing fees, sales taxes and other fees. These mark-ups also apply at different stages of the supply system in the private sector. Ball et al. [33] found that these mark-ups cumulatively equalled 8.7–47.7% of the manufacturer’s selling price for locally produced insulin and 10.0%–565.8% for imported insulins. It is important to note that, beyond price, a variety of geographical and health system factors can have an impact on access to insulin, such as distance of the patient to healthcare facilities [31].

### Prescribing

The prescribing of insulin is affected by the organisation of care and where in the health system (e.g. in the hospital or in primary healthcare [PHC]) people can receive their diabetes care. For example, in Vietnam, access to insulin is mainly provided in major urban areas and in hospitals [34]. In some contexts, only specialists are able to prescribe insulin [30]. At PHC level, there is often a lack of experience and expertise, gaps in knowledge, fear of hypoglycaemia, missing guidelines and even fear of prescribing insulin [35, 36]. Additional challenges to insulin prescribing include the fact that some health professionals no longer know how to use human insulin as they have received their training in contexts where only analogue insulin is used [37] and the possible impact that the pharmaceutical industry might have on prescribing decisions of doctors [38, 39].

### Dispensing

**Fig. 3:**
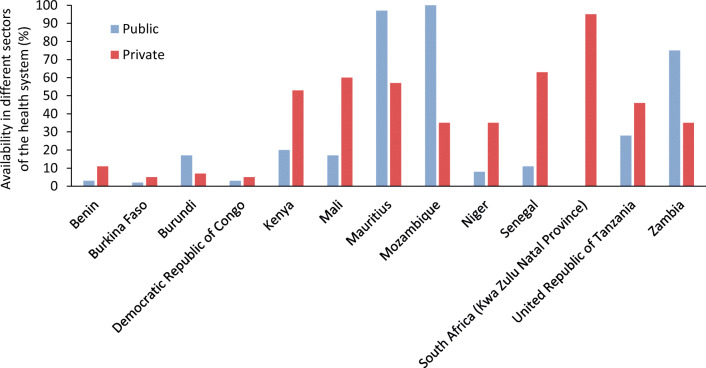
Availability of insulin in the public and private sectors in a variety of countries in sub-Saharan Africa. Availability was measured as insulin being present in a facility (public or private) on the day of the study visit. Data derived from [41–45]. This figure is available as part of a downloadable slideset

Dispensing, or the provision of insulin by the health system to an individual, faces two clear challenges in many LMICs [30, 31, 34]: (1) the often-large distance between communities and health facilities that provide diabetes care; and (2) the fact that insulin prescriptions may only be available from secondary or even tertiary healthcare facilities. Beyond these factors, there is also the issue that the insulin may not be available in the pharmacy when the individual goes to get it. For example, in Peru, although insulin is supposed to be available in the public sector, it is frequently unavailable, resulting in people going to the private sector, where insulin is more expensive [40]. Data from a variety of countries show that median patient prices for human insulin in the private sector are 2.2 times higher than in the public sector (Fig. 2). In addition, in 13 countries in sub-Saharan Africa [41–45], poor availability of insulin has been found in both the public and private sectors (Fig. 3), as well as at different levels of the health system, with, for example, insulin being more highly available in hospitals than in PHC settings. The WHO’s global action plan on noncommunicable diseases (NCDs) [46] comprises a target of 80% availability of essential medicines, which includes insulin; of the 13 countries studied in sub-Saharan Africa [41–45], only two met this target in the public sector and one met it in the private sector (Fig. 3).

### Insulin use

As noted by Holden et al. [47], in 1991, in the UK most insulin was used by people with type 1 diabetes. The use of insulin has increased globally, mainly due to the rising prevalence of type 2 diabetes [8, 47], and will continue to do so [3]. With this overall increase in use, a parallel increase in the use of analogues has also been seen [6]. High insulin prices mean that some people are not able to afford insulin and, therefore, either forego or economise their insulin use by not taking a full dose [6, 48]. Use of insulin is also influenced by the patient’s socioeconomic status, poor knowledge of diabetes, traditional beliefs, use of traditional medicine [35] and misperception of insulin as a ‘last resort’ therapy [49]. Basu et al. [3] also highlight that overuse of insulin can have a negative impact on disability-adjusted life years, especially in older populations.

## Beyond insulin

Since insulin needs to be injected, access to syringes or other delivery devices are essential for insulin therapy and are often not available and affordable [30]. Availability and affordability of diagnostic tests in healthcare facilities and glucose meters for self-monitoring of blood glucose was found to be poor in many LMICs [30, 50]. Ogle et al. [51] looked at a variety of LMICs and found that the annual median cost for the management of diabetes (insulin, blood glucose meters, test strips, syringes and HbA_1c_ tests) was US$533 (range: US$255 in Pakistan to US$1185 in Burkina Faso). Beyond these tools, a variety of health system factors are needed for the management of diabetes and these are particularly lacking in LMICs. The health system response for NCDs (including diabetes) proposed by the WHO focuses on PHC [46] and lacks a clear focus on access to medicines and health products and overall integrated health system responses [52]. In addition, other barriers to diabetes care are present, such as lack of healthcare worker training and shifting certain roles in diabetes management from doctors to nurses and other healthcare professionals, ill-adapted organisation of care, poor availability of tailored patient education materials and approaches relevant to the contexts and realities of people living in LMICs, lack of an active role of national diabetes associations in shaping diabetes care and policy, and lack of overall resources allocated to diabetes [53]. These various factors that impair diabetes care result in a low or decreased life-expectancy for people with type 1 diabetes [42, 48] and a gap in access to insulin for people with type 2 diabetes, which, in turn, can lead to excess morbidity and mortality.

## Summary

**Fig. 4:**
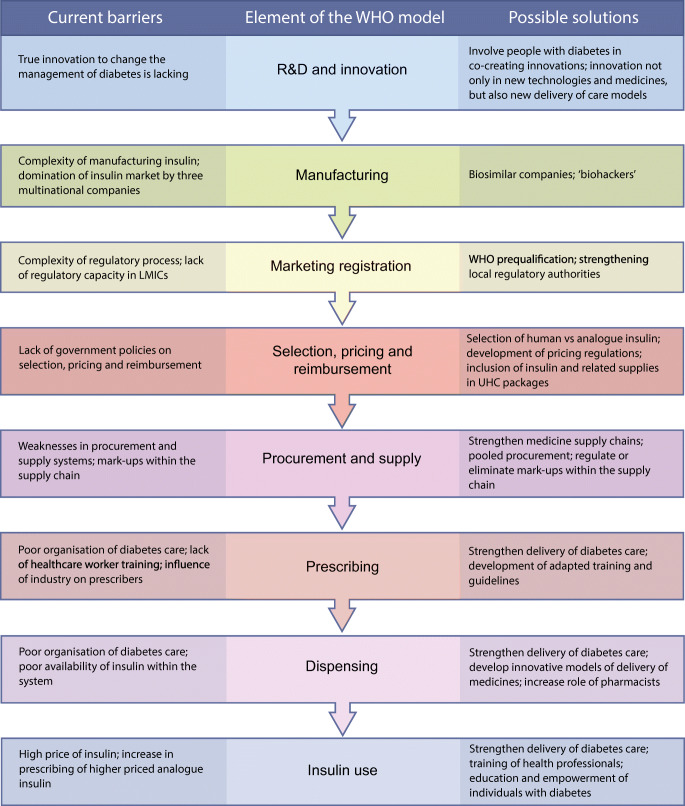
Summary of barriers and solutions to overcome these to improve access to insulin. This figure is available as part of a downloadable slideset

Access to insulin is truly a global challenge, with a wide range of issues that impact on an individual’s capacity to find and afford the insulin they need for their survival (Fig. 4). The fact that the insulin market is concentrated by three multinational companies affects the global market and has ramifications at a country level [6] . True innovation is lacking with regard to insulin and the delivery of diabetes care [14]. Regulatory requirements for insulin need to be stringent enough to protect the population from substandard medicines without being a barrier to market entry. The inclusion of insulin on the WHO Prequalification Programme [23] should be seen as a major advance in improving access to insulin in LMICs by allowing other insulin manufacturers entry into the market.

Insulin provides an interesting example of how evidence has not been adequately used to guide clinical practice. Despite analogue insulin not being included in the WHO Model EML, its uptake has been substantial despite its higher price [6, 14, 24]. This change in practice has an impact on the cost of diabetes management for both the individual and the health system. In addition, pricing policies and mark-ups within the health system and supply chain affect the overall financial burden of diabetes management [6, 30, 33].

Beyond the actual access to insulin, a variety of health system factors further hamper access to care; these include diabetes care being provided at hospitals vs in PHC, lack of knowledge and expertise of health professionals with regard to insulin therapy, and availability and affordability of diabetes-related supplies [30, 34, 40]. These health system factors can also have an impact on the overall cost of diabetes management, with individuals needing to pay for travel from their home to a facility where insulin is present or only being able to access this medicine in the private sector at higher prices [5, 30, 34, 40]. Although donation programmes have shown some success in demonstrating that, if access to insulin is improved, type 1 diabetes does not need to be a death sentence in LMICs, these programmes need to be better integrated into the health system [32].

At a global level, the United Nations’ sustainable development goals (SDGs) [54] include targets on NCDs, access to medicines and universal health coverage (UHC), which should guide the global response to diabetes [46]. For type 1 diabetes, many countries do not necessarily provide full coverage for insulin and diabetes supplies within the context of UHC [55]; should these be provided in a comprehensive way, this could have an impact on mortality [56]. To date, the global response to diabetes has mainly focused on prevention rather than diabetes care and access to medicines [57]. Both globally and nationally, a lack of leadership, civil society mobilisation and guiding institutions has meant that the issue of access to insulin has not been comprehensively addressed [7]. The launch of the Global Diabetes Compact by WHO in November 2020 [58] is a positive step; however, the content and approach of this initiative still needs to be defined.

All solutions for improving access to insulin (Fig. 4) need to have people with diabetes playing a key role, both being driven by people with diabetes and being designed with people with diabetes in mind. They must also include innovations that decrease global inequalities. Different players will need to be involved, including the WHO, diabetes associations, academia and the private sector. International support is needed to advance WHO prequalification, strengthen national regulatory agencies, and improve processes surrounding the selection, pricing, reimbursement, procurement and supply of insulin. For these aspects, the WHO plays a crucial role in supporting its member states. The WHO or other organisations within the United Nations can also develop a pooled procurement mechanism for insulin and diabetes management-related supplies. In addition, global clinical and scientific diabetes societies have a role in the development of context-appropriate and conflict-of-interest-free guidelines and training. National governments can develop comprehensive policies on selection and pricing of products for diabetes management, ensure insulin and other diabetes supplies are included in UHC packages and remove or regulate mark-ups within the supply chain. Overall, health systems and the way in which diabetes care is delivered needs to be strengthened, ensuring that it is centred on the needs of people with diabetes.

The private sector has a clear mandate, which is included in the SDGs and can play a role in addressing the challenge of access to insulin. Initiatives should focus on creating true partnerships with other players (as is the case in other areas of health [59]) and addressing the issue of the price of insulin in a sustainable way. As aforementioned, one area that needs to be further strengthened at a national and global level is civil society mobilisation. Although global and national diabetes organisations exist and provide training, guidelines and education, so far, they have failed to materialise into a truly global movement and ensure that the voice of people with diabetes is heard. The centenary of insulin offers a policy window for this to happen and shape the future of access to insulin and diabetes care globally.

## Supplementary Information

Slideset of figures (PPTX 360 kb)

## Abbreviations

EML: Essential Medicines List
LMIC: Low- and middle-income country
NCD: Noncommunicable disease
PHC: Primary healthcare
R&D: Research and development
SDG: Sustainable development goal
UHC: Universal health coverage
