# Supplementary material for: A global perspective on the issue of access to insulin
Source: Diabetologia. 2021 Jan 23;64(5):954–62. doi: 10.1007/s00125-020-05375-2 (PMC8012321; doi:10.1007/s00125-020-05375-2)
Supplement: Supplementary file 1 — (PPTX 360 kb) [file 125_2020_5375_MOESM1_ESM.pptx]

## Slide 1
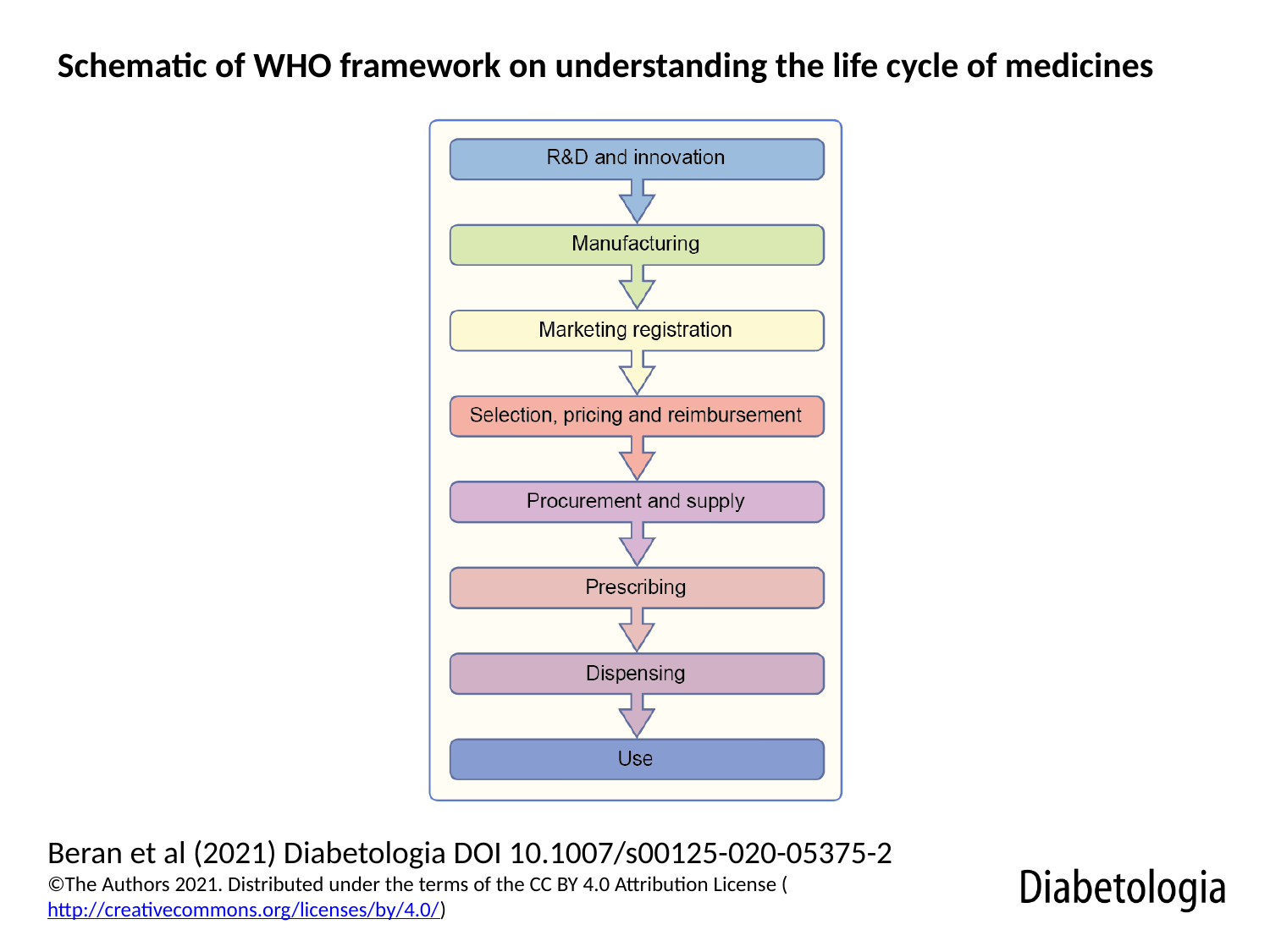

Schematic of WHO framework on understanding the life cycle of medicines
Beran et al (2021) Diabetologia DOI 10.1007/s00125-020-05375-2
©The Authors 2021. Distributed under the terms of the CC BY 4.0 Attribution License (http://creativecommons.org/licenses/by/4.0/)

## Slide 2
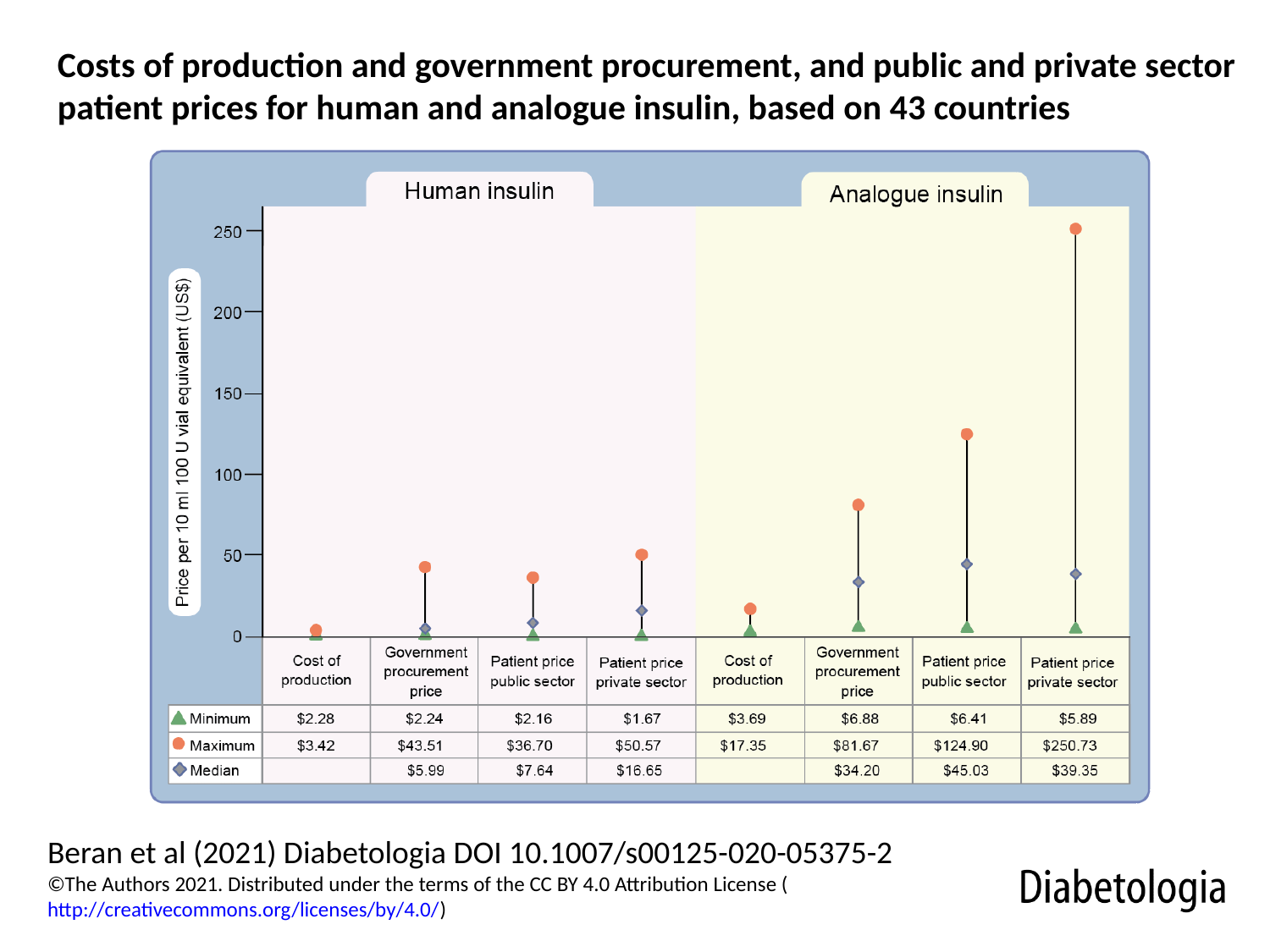

Costs of production and government procurement, and public and private sector patient prices for human and analogue insulin, based on 43 countries
Beran et al (2021) Diabetologia DOI 10.1007/s00125-020-05375-2
©The Authors 2021. Distributed under the terms of the CC BY 4.0 Attribution License (http://creativecommons.org/licenses/by/4.0/)

## Slide 3
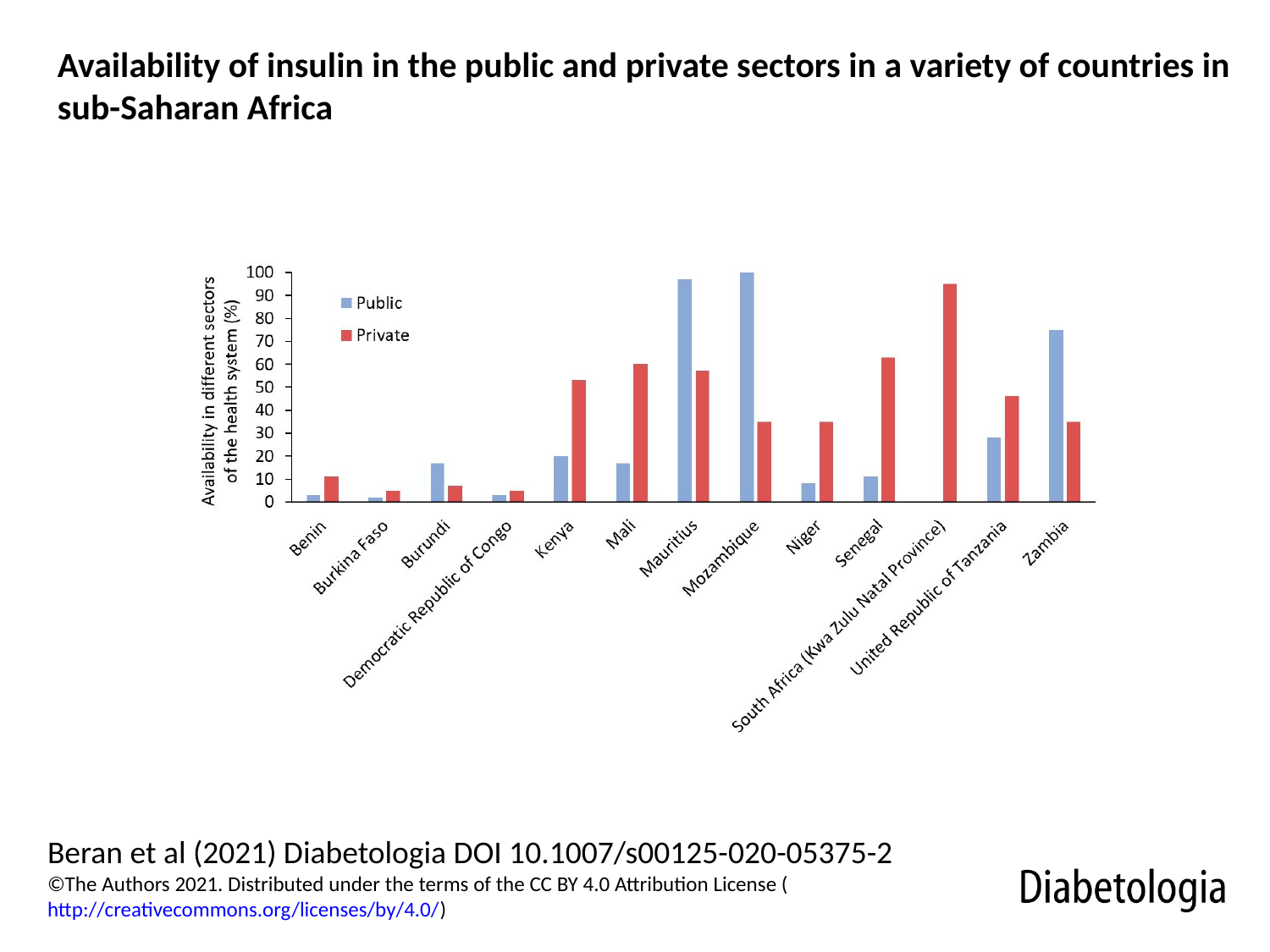

Availability of insulin in the public and private sectors in a variety of countries in sub-Saharan Africa
Beran et al (2021) Diabetologia DOI 10.1007/s00125-020-05375-2
©The Authors 2021. Distributed under the terms of the CC BY 4.0 Attribution License (http://creativecommons.org/licenses/by/4.0/)

## Slide 4
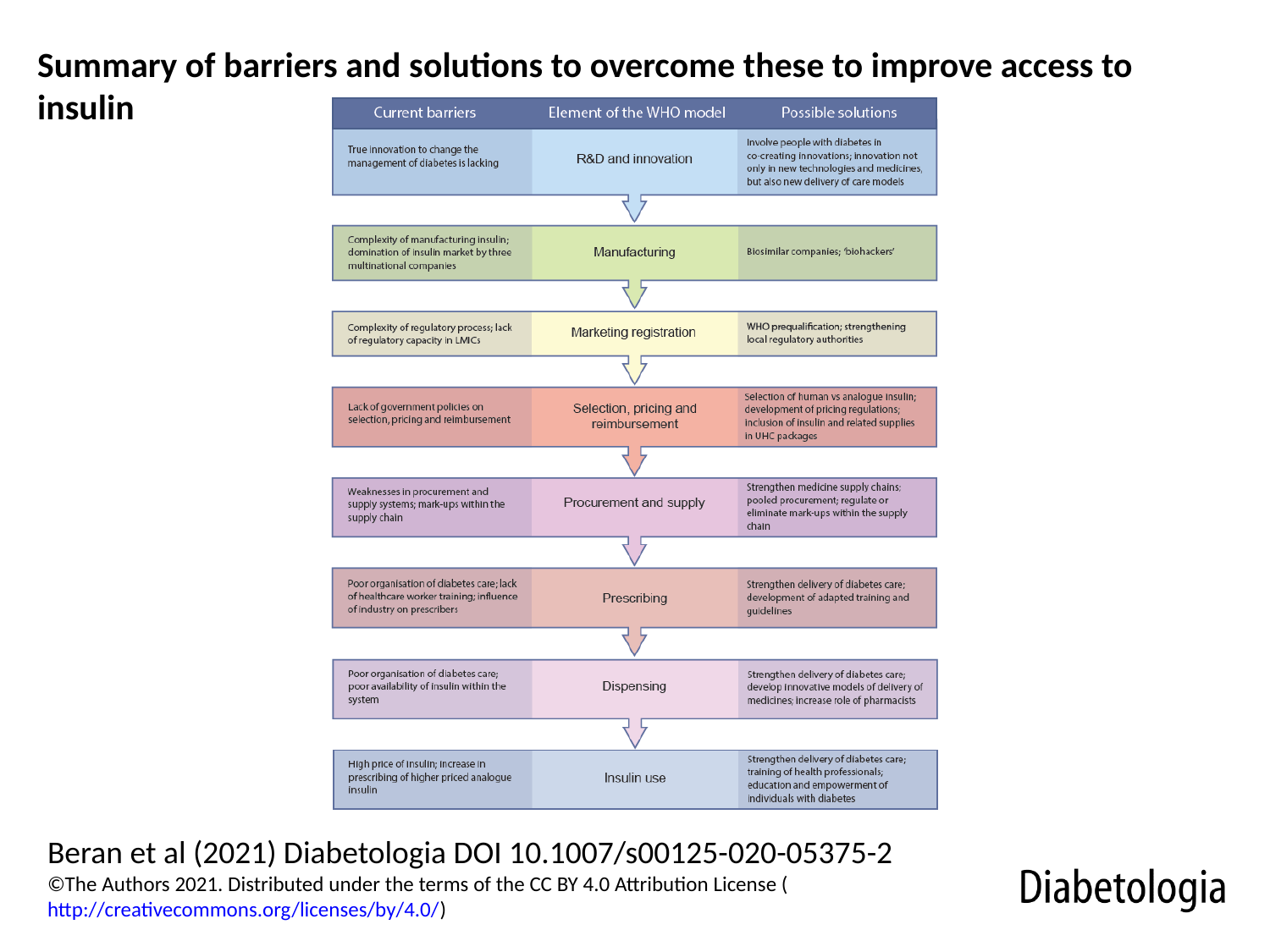

Summary of barriers and solutions to overcome these to improve access to insulin
Beran et al (2021) Diabetologia DOI 10.1007/s00125-020-05375-2
©The Authors 2021. Distributed under the terms of the CC BY 4.0 Attribution License (http://creativecommons.org/licenses/by/4.0/)
